# Concurrent pulmonary tuberculosis and lepromatous leprosy in a newly diagnosed HIV positive patient: a case report

**DOI:** 10.1186/s12890-021-01572-w

**Published:** 2021-06-30

**Authors:** Josiah T. Masuka, Zamambo Mkhize, Somasundram Pillay, Anisa Mosam

**Affiliations:** 1 Department of Dermatology, Nelson R Mandela School of Medicine, Private Bag X7, Congella, Durban, 4013 South Africa; 2 Department of Dermatology, Edendale Hospital, 89 Selby Msimang Rd, Pleissislaer, Pietermaritzburg, 3201 South Africa; 3 Harare Central Hospital, PO Box ST14, Southerton, Harare, Zimbabwe; 4 Department of Internal Medicine, Nelson R Mandela School of Medicine, Private Bag X7, Congella, Durban, 4013 South Africa

**Keywords:** Leprosy, Pulmonary tuberculosis, Syphilis, HIV, Coinfection

## Abstract

**Background:**

The leprosy-tuberculosis (TB) co-infection is rarely reported in recent times. However, this dual comorbidity is associated with high mortality and major morbidity. Unrecognised leprosy-TB co-infection may predispose affected patients to rifampicin monotherapy and subsequent drug resistance.

**Case presentation:**

A 35 year old migrant, human immunodeficiency virus (HIV) positive male worker presented with 6 month history of symmetric infiltrative nodular plaques of the face and distal, upper extremities. A few days after initial dermatology presentation, a sputum positive pulmonary tuberculosis diagnosis was made at his base hospital. Subsequent dermatology investigations revealed histology confirmed lepromatous leprosy and a weakly reactive rapid plasma reagin test. The presenting clinical features and laboratory results were suggestive of lepromatous leprosy coexisting with pulmonary tuberculosis in an HIV positive patient.

**Conclusions:**

This case illustrates the occurrence of leprosy with pulmonary tuberculosis in an HIV infected patient and the difficulties in interpreting non-treponemal syphilis tests in these patients. This case also highlights the need for a high index of suspicion for co-infection and the need to exclude PTB prior to initiation of rifampicin containing multi-drug therapy (MDT). Interdisciplinary management and social support are crucial in these patients.

**Supplementary Information:**

The online version contains supplementary material available at 10.1186/s12890-021-01572-w.

## Background

Tuberculosis (TB) and leprosy are chronic granulomatous infectious diseases resultant from aerosol spread of the intracellular gram positive, aerobic bacilli - *Mycobacterium tuberculosis* and *Mycobacterium leprae* respectively. Both diseases are of public health importance as they cause significant morbidity and mortality. In addition, there are concerns of drug resistance especially for *M. tuberculosis* [1]. These infections share similar transmission dynamics and are more prevalent in low socio-economic settings characterized by overcrowding, poor sanitation and malnutrition [2–4]. A significant proportion of the population in Asia and Africa are latently infected by *M. tuberculosis* and/or *M. leprae*, however only around 5 % of these infected individuals develop overt clinical TB or leprosy [4]. Both diseases show polarity ranging from pauci-bacillary to multi-bacillary disease depending on the host’s cell mediated immune response [5]. Unlike TB, the level of host systemic immunosuppression due to HIV infection does not appear to significantly modify the natural course and/or the clinical presentation of leprosy [6–8]. However, the immune reconstitution inflammatory syndrome (IRIS) can complicate the management of both infections in patients on antiretroviral treatment [6, 7].

HIV has rarely been reported in a patient co-infected with pulmonary tuberculosis (PTB) and nodular leprosy [9]. Infrequently, nodular syphilitic plaques have been misdiagnosed as lepromatous, tuberculoid or borderline leprosy in HIV positive [10–12] as well as immunocompetent patients [13, 14]. On the other hand patients with leprosy may present with biologically false positive (BFP) results; conversely 16 % of leprosy patients with BFP results have treponemal disease suggestive of present or past syphilis infection [15]. In this case report, we describe a newly HIV diagnosed patient co-infected with nodular appearing, histologically confirmed lepromatous leprosy, pulmonary TB (PTB) and suspected secondary syphilis. This case highlights the diagnostic challenges during patient work-up and the therapeutic challenges encountered in the patient’s PTB treatment given the previously undiagnosed leprosy.

## Case presentation

A 35 year old Malawian single, self-employed immigrant presented as a referral to the Edendale hospital dermatology clinic for non-pruritic rash on his face, forearms and hands. He had also been referred to our hospital’s ophthalmology clinic for a corneal opacification in his left eye. The rash had been present for the past 6 months. On his index dermatology consultation, it was noted that he had been recently diagnosed and initiated on highly active antiretroviral therapy (HAART) - a week prior to the visit. The initial CD4 count and viral load were not available from his base hospital. History revealed that at the time of HIV diagnosis, he had presented to his base hospital with a generalized illness associated with malaise, night sweats and fever without an associated cough. Unbeknown to our dermatology team, in the week after the initial dermatology consult, he was subsequently started on a 6 month course of anti-tubercular medications for GeneXpert MTB/Rif Ultra® sputum positive PTB sensitive to rifampicin at his base hospital. The rest of his past medical and surgical history was unremarkable. He reported that he occasionally traveled to visit his family in Malawi and that his last visit was 3 years previously.

**Fig. 1:**
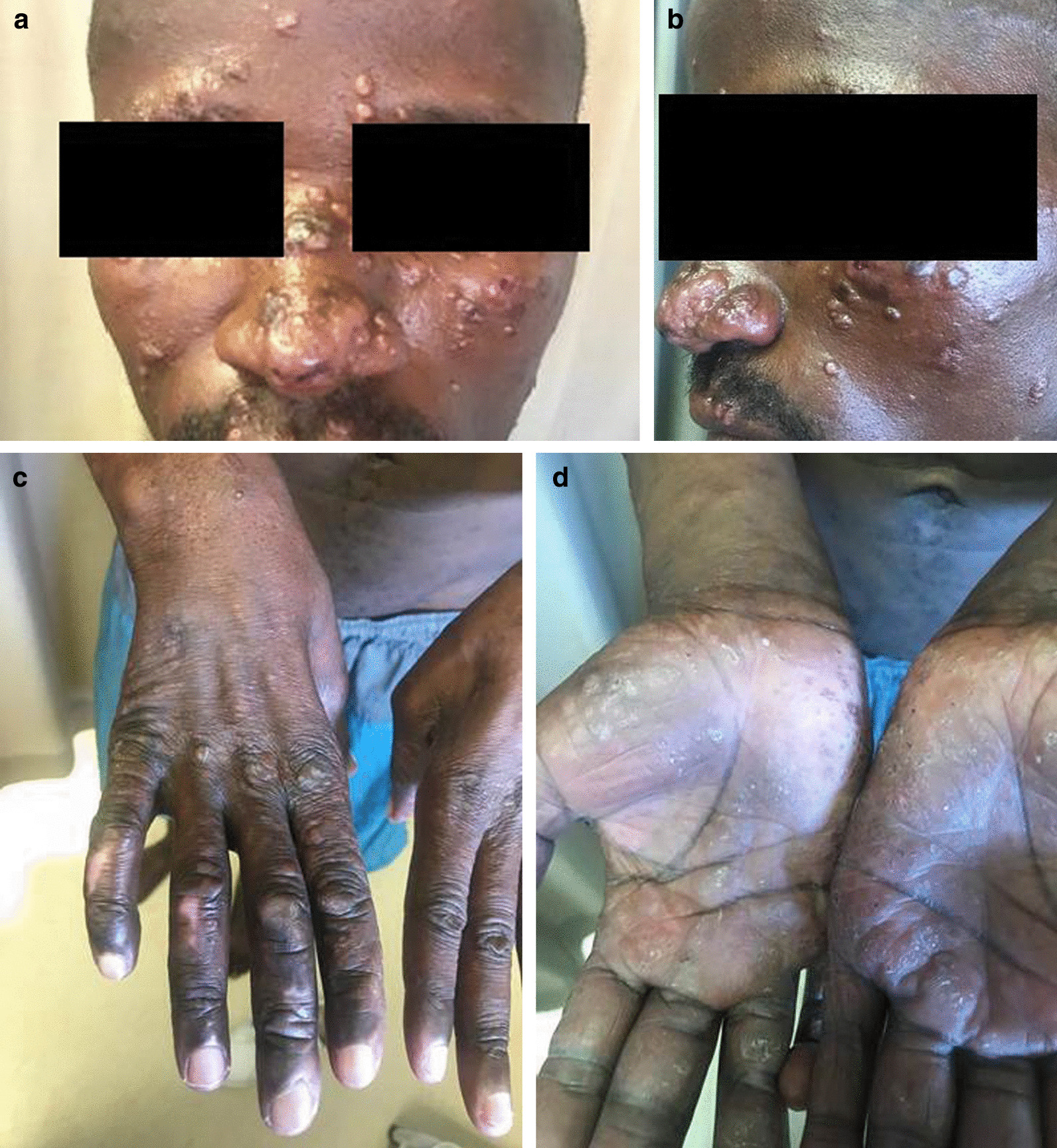
Clinical images of the facial (**a**, **b**) and hand lesions (**c**, ** d**) on initial presentation

On examination, the patient had symmetric infiltrated skin coloured plaques and nodules on the nasal bridge, the forehead, the forearms and the dorsum of both hands as shown in Fig. 1. The palms of both hands displayed a keratoderma characterized by brownish plaques and there was a healed burn scar on the dorso-lateral surface of the right hand. An appreciable flexion deformity of the right little finger was also observed. A left eye corneal opacification accompanied by bilateral episcleritic red eyes was also evident. Apart from the keratoderma, no other stigmata of secondary syphilis were observed. The patient had a normal gait and the rest of the examination was unremarkable. A 5 mm punch biopsy of his forehead lesions along with blood tests were performed as indicated in Additional file 1: Table 1. Our initial differential diagnosis of the infiltrative nodulo-papular plaques included amongst others sarcoidosis, leprosy, syphilis, deep fungal infections and scleromyxoedema [16]. His serum angiotensin converting enzyme (SACE) result was 91U/L (range 20.0–70.0U/L) and whilst awaiting histology results our primary diagnosis was sarcoidosis. We started the patient on topical dovate ointment, chloroquine 200 mg/day. On his next review, a weakly reactive RPR result with a titre of 1:2 dilutions was noted. As the World Health Organisation (WHO) recommended benzathine penicillin was not available, the patient was initiated on ceftriaxone 1 gram intramuscularly stat and doxycycline 100 mg bd per oral daily for 1 month.

**Fig. 2:**
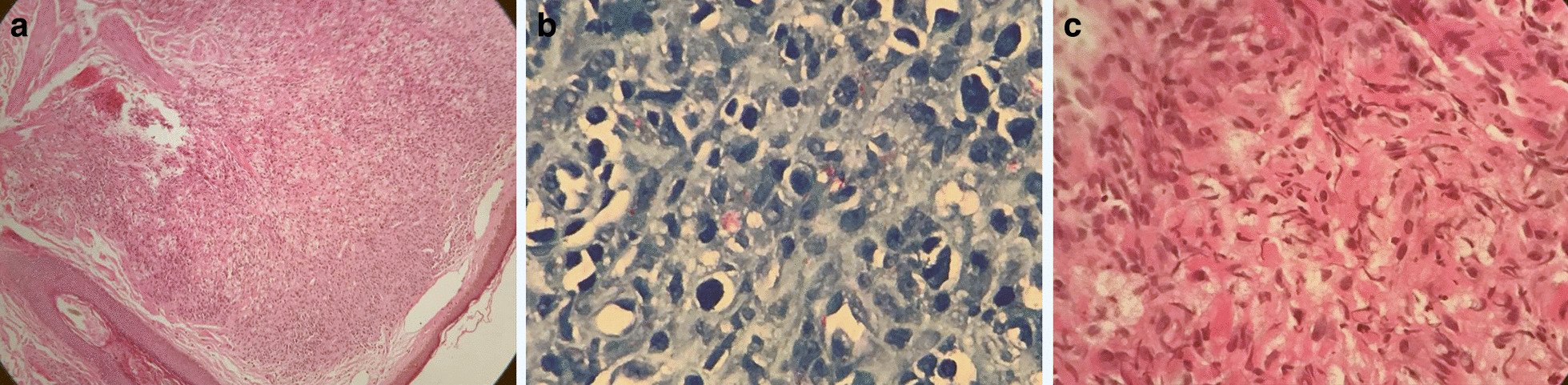
Histopathology images of the facial lesions: hematoxylin and eosin stain showing the granulomatous inflammation in the dermis (**a**) and fite stain demonstrating mycobacterium leprae bacilli (**b**, **c**)

On his third review, 2 months after initial presentation, we noted that the forearm lesions had disappeared while the facial plaques were significantly regressing. The patient’s RPR was no longer reactive and his biopsy results revealed a histopathological diagnosis of lepromatous leprosy as shown in Fig. 2. This prompted a re-examination of his neurological system which revealed glove and stocking loss of sensation in both hands and feet respectively. The posterior tibial and radial nerves were also noted to be palpable in the upper and lower limbs respectively. Our patient was started on the WHO recommended MDT for leprosy. A month later, he experienced worsening of his facial lesions which led us to believe that the combination of ceftriaxone and doxycycline had been more efficacious than the current therapy. A paradoxical, leprosy associated IRIS reaction and a type I reversal leprosy reaction were considered in the differential diagnosis of the worsening facial plaques. Both present similarly making a diagnosis difficult [7], however, in our patient, the type I reversal reaction was favoured over a leprosy-associated IRIS reaction in the current case report as the later did not meet the specified definition for the condition [7]. Unfortunately, the patient was not re-biopsied and no specific treponemal test such as TPHA was done.

## Discussion and conclusions

We have presented a case of concurrent lepromatous leprosy and PTB in an HIV positive patient with suspected secondary syphilis. HIV, PTB and leprosy coinfection has not been reported in the literature [17]. However, a case of reactivated cutaneous TB and leprosy in an HIV patient has been reported [9]. Similarly, a few cases of leprosy coinfection with syphilis in an HIV positive patient have previously been reported [18–20]. Meanwhile, *M. tuberculosis* and *M. leprae* coinfection has rarely been reported in the same patient even in countries where both diseases are endemic, but it has increased mortality and morbidity [4, 5, 21–23]. The reported leprosy-TB coinfected cases presented with pulmonary and/or extra-pulmonary TB—cutaneous, lymph nodal, perianal and peritoneal lesions [4, 21, 23–27]. Speculative hypotheses have indicated that leprosy, especially the anergic/pauci-bacillary form predisposes to TB whereas others have postulated an antagonistic relationship between the two mycobacterial infections [5]. Leprosy-TB coinfection is rare possibly due to the different mycobacterial reproduction rates and the possible cross-immunity between the two organisms [24, 28]. In the last 15 years, the leprosy-TB coinfection case reports have been described in the setting of systemic corticosteroids for a lepromatous type I reversal reaction in a patient initially diagnosed as either borderline or lepromatous leprosy [21]. In most leprosy-TB coinfections, TB follows leprosy infection [23, 29]. An undiagnosed leprosy-TB coinfected patient runs the risk of receiving rifampicin monotherapy and the subsequent development of rifampicin-resistant TB during leprosy therapy [21, 23]. Sputum and a chest radiograph to exclude PTB are required in the initial work-up of a patient with leprosy [21]. Furthermore, the substitution of minocycline for rifampicin whilst awaiting the TB screening results, directly observed therapy and intensive medical monitoring may be necessary to prevent poor treatment outcomes [21, 23].

Both leprosy and syphilis were considered in the clinical differential diagnosis of our patient’s infiltrative plaques. However, leprosy and syphilis may present a challenging differential diagnosis [19]. Both diseases have polymorphous presentations and occasionally present unclear laboratory tests which hinder definitive clinical diagnosis of the presenting lesions [14, 19]. Syphilis,” the great imitator” [30], normally presents as a papulo-squamous rash, but in HIV positive patients it may present atypically as annular, nodular or granulomatous plaques [10, 14]. In fact, nodular and granulomatous syphilis has occasionally been clinically and/or histopathologically misdiagnosed as indeterminate [31] or tuberculoid [10] or borderline [11, 12, 14] lepromatous [32] leprosy especially in HIV infected patients [10]. Given the aforementioned confusion in earlier cases even on histology, the weakly reactive RPR result and the initial improvement on ceftriaxone and doxycycline, syphilitic palmar keratoderma and ocular lesions [33] remained a possible diagnosis in our patient. The initial improvement following the institution of ceftriaxone and doxycycline for the suspected syphilis was probably just due to rifampicin monotherapy for leprosy whilst the patient was receiving PTB treatment from his base hospital. Whilst this regimen is effective for early syphilis [34], it is not effective for leprosy. Of the tetracyclines, only minocycline is effective for leprosy [35].

Even though our patient was HIV positive, his HIV status neither increased his risk for leprosy nor for lepromatous leprosy as previously feared [7]. In fact, HIV does not alter the clinical, immunological nor histopathological spectrum of leprosy features even in patients with advanced immunodeficiency [8, 19, 20]. In fact, upgrading reactions characterized by organized granulomas and a competent cellular immune response involving activated macrophages, CD4+ and CD8+ cells have been described in leprosy-HIV coinfected patients [36]. Borderline and tuberculoid leprosy presentations may actually be more common in HIV infected compared to HIV uninfected patients [7, 36, 37]. However, leprosy may aggravate HIV infection though data is limited in this regard [5, 7]. These observations suggest that cell-mediated immune responses are preserved at the site of *M. leprae* infection whereas they are abrogated systemically in contrast to TB [8]. Leprosy co-infection with either HBV, HCV, HTLV-1 is associated with increased risk for inflammatory and nerve damage complications as well as leprosy relapse [38]. Furthermore, immune dysregulation with exacerbation of either disease may result in the HIV-leprosy patient coinfected with a third infectious pathology such as syphilis [20]. Moreover, HIV positive patients have an increased risk for leprosy type I reversal reactions and paradoxical leprosy-IRIS especially when they are on HAART [5, 7, 8]. Thus, stricter monitoring is needed in HIV-leprosy coinfected patients compared to those without HIV. On the other hand, HIV and syphilis coinfection occurs more frequently as both infections affect a similar patient profile [20]. In addition, syphilis increases the chances of HIV transmission and/or infection [39]. In HIV positive patients, syphilis often presents atypically, with shortened time between primary and disproportionately aggressive secondary syphilis [20, 39]. Thus, it is always prudent to include syphilis in the differential diagnosis of any rash and especially papulo-nodular rashes in an HIV positive patient [16].

However, several case reports have indicated that treponemal specific tests may have high false negative results whereas non-treponemal tests may can have high rates of false positive, non-falling results or falsely non-reactive [33]. Similarly, the weakly reactive RPR not only suggested syphilis, it could simply have been a biologically false positive (BFP) result due to leprosy, TB and/or HIV infection [30, 39]. Lepromatous and borderline leprosy are highly associated with BFP results [40]. There is a distinct possibility that the initial partial leprosy treatment with rifampicin reduced the chance of an RPR BFP result on retest in our patient. Studies have demonstrated that leprosy patients may have an inordinately higher frequency of concurrent syphilis, though the results supporting this notion still require more analysis for verification [41–43]. BFP results have also been noted to be significantly higher in HIV infected individuals compared to non-HIV infected individuals [44]. However, HAART appears to decrease the odds of biologically false positive RPR results in HIV infected individuals [41, 45]. Confusingly, false-negative syphilis results have also been noted in primary and secondary syphilis [39]. Moreover, our patient’s raised SACE levels could also be explained by leprosy, sarcoidosis, TB as well as HIV infection amongst a host of other diseases [46].

In conclusion, clinicians ought to be aware that leprosy and PTB may occur concurrently and they may need to rule out one if the other is present [21]. A thorough history and examination coupled with good clinico-pathological correlation is necessary in diagnosing atypical or unusual dermatoses [12]. Undiagnosed leprosy-TB coinfection runs the risk of rifampicin monotherapy which subsequently increases the chances of developing resistant mycobacterial strains [21, 47]. In this and other HIV positive patients, it is important to screen and start treatment for PTB before the institution of HAART in accordance with the guidelines to prevent PTB IRIS reactions [48, 49]. In addition, all pts with HIV should be screened for syphilis and vice versa [39]. Furthermore, a treponemal antibody test is required in a patient with leprosy to disprove a reactive non-treponemal test such as an RPR [41, 43]. Leprosy patients with both reactive treponemal and non-treponemal syphilis tests should be treated for syphilis [41]. Though not significantly relevant in the presented case, in migrant populations, it is prudent to review RPR results in light of the endemic treponemal diseases in their home country so as not to miss other treponemal diseases such as yaws and pinta. Finally, there is need for better communication on patient management between primary and referral hospitals to optimize therapeutic outcomes and prevent mismanagement. Both infections should be notified.

## Supplementary Information

**Additional file 1**. **Table 1:** Tabulated laboratory results.

## Data Availability

The datasets used and/or analysed during the current study are available from the corresponding author on reasonable request.

## Abbreviations

CD: Cluster of Differentiation
CRP: C Reactive Protein
ESR: Erythrocyte Sedimentation Rate
HAART: Highly Active Anti-retroviral Therapy
HIV: Human Immunodeficiency Virus
IRIS: Immune Reconstitution Inflammatory Syndrome
MDT: Multi-drug Therapy
RPR: Rapid Plasma Reagin
PTB: Pulmonary Tuberculosis
VDRL: Venereal Diseases Research Laboratory
WHO: World Health Organisation
