# Supplementary material for: Concurrent pulmonary tuberculosis and lepromatous leprosy in a newly diagnosed HIV positive patient: a case report
Source: BMC Pulm Med. 2021 Jun 30;21:207. doi: 10.1186/s12890-021-01572-w (PMC8247149; doi:10.1186/s12890-021-01572-w)
Supplement: Supplementary file 1 — Additional file 1. Table 1: Tabulated laboratory results. [file 12890_2021_1572_MOESM1_ESM.docx]

**Supplementary Table 1**: Tabulated laboratory results

| **Date** | **Test** | **Parameter** | **Value** | **Range** |
| --- | --- | --- | --- | --- |
| Initial | Urea & electrolytes Ca^2+^, Mg^2+^ & inorganic phosphate | all normal |  |  |
|  | Liver function tests | All normal |  |  |
|  | TB test | Gene Xpert MTB/Rif Ultra | M. tuberculosis detected | Rif sensitive |
| 6 months later | Full Blood Count | haemoglobin | 14.1 | 13.0 – 17.0 |
|  |  | leucocytes | **2.46** | 3.92 – 10.40 |
|  |  | platelets | 177 | 171 - 388 |
|  |  | MCV | 95.2 | 83.1 – 101.6 |
|  |  | Neutrophils | **1.13** | 1.60 – 6.98 |
|  |  | Lymphocytes | **0.73** | 1.40 – 4.20 |
|  |  | Monocytes | 0.37 | 0.30 – 0.80 |
|  |  | Eosinophils | 0.19 | 0.00 – 0.95 |
|  |  | Basophils | 0.04 | 0.00 – 0,.10 |
|  | TB test - sputum | Auramine O stain | No AFB/100 immersion fields |  |
|  | CD45+ WCC |  | 6.92 x10^9^/L | 4.00 – 10.00 |
|  | CD4% |  | 42.22% | 28.00 – 51.00 |
|  | AbsoluteCD4 count |  | 412 | 332 - 1642 |
|  | Viral load |  | Undetectable |  |
|  | Non-treponemal syphilis test | RPR | Non - reactive | - |

**WCC – white cell count; MCV – mean corpuscular volume
